# Cassie’s Law Reformulated: Composite Surfaces from Superspreading to Superhydrophobic

**DOI:** 10.1021/acs.langmuir.3c01313

**Published:** 2023-07-24

**Authors:** Glen McHale, Rodrigo Ledesma-Aguilar, Chiara Neto

**Affiliations:** † Institute for Multiscale Thermofluids, School of Engineering, The University of Edinburgh, Edinburgh EH9 3FB, U.K.; ‡ School of Chemistry and the University of Sydney Nano Institute, The University of Sydney, Sydney, New South Wales 2006, Australia

## Abstract

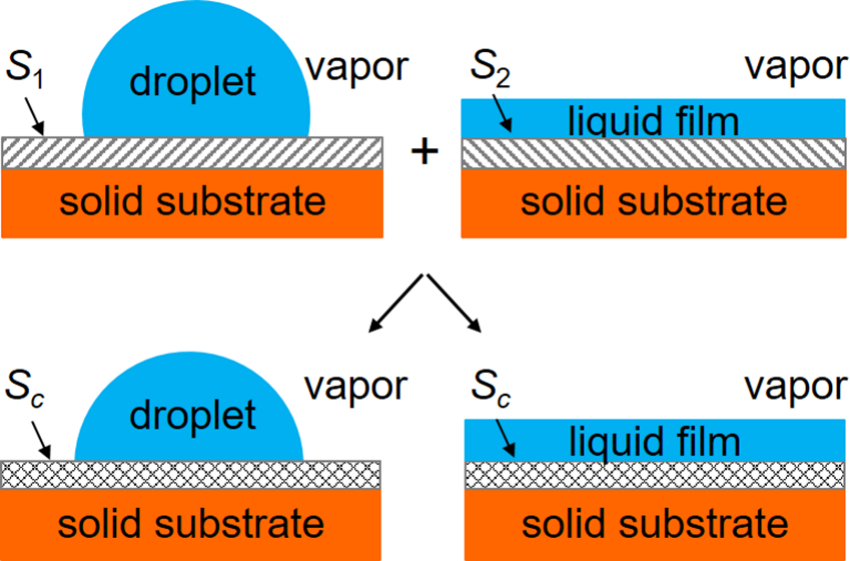

In 1948, Cassie provided an equation describing the wetting of a smooth, heterogeneous surface. He proposed that the cosine of the contact angle, θ_c_, for a droplet on a composite surface could be predicted from a weighted average using the fractional surface areas, *f*_*i*_, of the cosines of contact angles of a droplet on the individual component surfaces, i.e., cos θ_c_ = *f*_1_ cos θ_1_ + *f*_2_ cos θ_2_. This was a generalization of an earlier equation for porous materials, which has recently proven fundamental to underpinning the theoretical description of wetting of superhydrophobic and superoleophobic surfaces. However, there has been little attention paid to what happens when a liquid exhibits complete wetting on one of the surface components. Here, we show that Cassie’s equation can be reformulated using spreading coefficients. This reformulated equation is capable of describing composite surfaces where the individual surface components have negative (droplet state/partial wetting) or positive (film-forming/complete wetting) spreading coefficients. The original Cassie equation is then a special case when the combination of interfacial tensions results in a droplet state on the composite surface for which a contact angle can be defined. In the case of a composite surface created from a partial wetting (droplet state) surface and a complete wetting (film-forming) surface, there is a threshold surface area fraction at which a liquid on the composite surface transitions from a droplet to a film state. The applicability of this equation is demonstrated from literature data including data on mixed self-assembled monolayers on copper, silver, and gold surfaces that was regarded as definitive in establishing the validity of the Cassie equation. Finally, we discuss the implications of these ideas for super-liquid repellent surfaces.

## Introduction

Understanding the wettability of surfaces is important for both industrial applications, such as paints, printing, and automobiles, and for naturally occurring surfaces, such as plants and insects.^1^ Many of such surfaces are often heterogeneous due to variations in surface chemistry or surface topography, and this is often characterized in an idealized manner by the contact angle of a droplet, ignoring contact line pinning. For over 70 years, Cassie’s equation (sometimes referred to as Cassie’s Law) has been a fundamental conceptual contribution to understanding the average contact angle on smooth, heterogeneous surfaces.^2^ This equation for the contact angle, θ_c_, of a droplet on a smooth, heterogeneous surface composed of two materials of fractional surface areas *f*_1_ and *f*_2_, where the liquid forms droplets with contact angles θ_1_ and θ_2_, states^2^
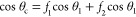
1

This equation is an extension of earlier work by Cassie and Baxter on the apparent contact angle on porous surfaces,^3^ which itself was an extension of ideas by Adam^4^ and Wenzel^5^ on apparent contact angles on rough surfaces. On a porous solvophobic surface, the droplet bridges across air gaps. One contact angle in eq 1 is then 180° and the surface area fractions *f*_1_ and *f*_2_ can be dependent on the contact angle on the solid surface.^3^ In the modern era of superhydrophobic surfaces, which commenced with the work of Onda et al.^6^ and Neinhuis and Barthlott,^7,8^ the Cassie–Baxter version of eq 1 has underpinned our understanding of surface wettability. This has often involved a simplified version used for patterned topographic surfaces with flat-topped micropillars, where
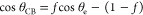
2Here, the Cassie–Baxter (suspended state) contact angle θ_CB_ is a weighted average using the solid surface area fraction, *f*, and Young’s law contact angle, θ_e_, given by
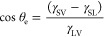
3where the γ_*ij*_ are the solid–vapor (SV), solid–liquid (SL), and liquid–vapor (LV) interfacial tensions. Equations 1 and 2 are only valid in an average sense when the droplet contact area is much larger than any characteristic length scale for patterns of the wettability of the solid surface, and when effects such as faceting and pinning at the boundaries of surface patterns can be ignored. The original works of Cassie and Wenzel tended to emphasize global averages for solid surface fractions and surface roughness. However, the literature has subsequently recognized that solid surface fractions and surface roughness *r* should be interpreted as values local to the three-phase contact line and so can be spatially varying, i.e., *f* = *f*(*x*) and *r* = *r*(*x*). For viewpoints on the appropriateness or otherwise of the Cassie model, see the discussion by Gao and McCarthy^9^ response by McHale,^10^ the recent paper by Shardt and Elliott,^11^ and the review by Quéré.^12^ The paper by Choi et al. also provides further discussion on the definition and calculations of solid surface fractions.^13^ The utility and importance of the concept of contact angle can also be extended to include slippery liquid-infused porous surfaces (SLIPS)^14,15^ and other liquid-infused surfaces^16,17^ where a droplet either rests entirely or partially on a lubricant layer.^18−22^

When eq 1 was first published in 1948 it was not clear whether it would be the most appropriate theory to describe droplet states on smooth composite surfaces. However, the work in 1992 by Laibinis and Whitesides on the wettability of composite surfaces created from mixed self-assembled monolayers adsorbed from solution onto surfaces of copper, silver, and gold films appeared to settle any debate.^23,24^ Recently, work by Becher-Nienhaus et al. on smooth checkerboard-like micropatterned hydrophobic/hydrophilic/complete wetting surfaces with regions of matching/mismatching contact angle hysteresis (CAH) has questioned whether eq 1 accurately describes experimental data.^25^ Their surfaces used four types of surface chemistry, encompassing hydrophobic (with low and high CAH), hydrophilic (with low CAH), and complete wetting properties for water droplets. In their analysis, these authors considered both composite surfaces created using two partial wetting surface chemistries and composite surfaces with one partial wetting and one complete wetting surface chemistries. In this latter case, they used eq 1 with the assumption that θ = 0° for the complete wetting surface component. This is unusual from the point of view of interfacial energies because once complete wetting occurs, there is no longer a well-defined equilibrium contact angle. Thus, to suggest different liquids giving complete wetting could all be characterized by a single contact angle of θ = 0° would be inconsistent, as discussed below.

For liquids spreading on ideal smooth flat solids in the presence of vapor, the spreading coefficient, *S*_LS(V)_, can be defined as the difference between the interfacial energy per unit area for a bare solid surface and one coated with a thin liquid film,^1,26^ i.e.,
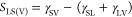
4In defining eq 4, we recognize that achieving ideal clean solid surfaces can be difficult experimentally due to contaminants and that estimates of the interfacial tension between a solid and a liquid are obtained via related equations, as discussed by Harkins and Feldman and others.^1,24,26^ From the lowest energy considerations, the spreading coefficient in eq 4 is negative for partial wetting droplets and greater than or equal to zero for liquid films. In this latter case, a range of surfaces, each with a different positive value of *S*_LS(V),_ may induce a liquid to spread, but it would not be reasonable to conclude that all correspond to a wetting state with θ = 0°. When the spreading coefficient is negative and a partially wetting droplet is observed, it is possible to write a relationship between the spreading coefficient and the contact angle
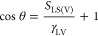
5

Thus, eq 5 defines a physical contact angle, θ, for an equilibrium droplet using the interfacial tensions via eq 4. By definition, the requirement for an equilibrium droplet shape restricts the cosine of the contact angle to the range −1 ≤ cos θ ≤ + 1. However, from an interfacial tension perspective, the right-hand side of eq 5 can have values that lead to a number for “cos θ” outside this restricted range, although a corresponding contact angle, θ, cannot then be calculated. For example, if the interfacial tensions combine in eq 4 to give a spreading coefficient which is positive, eq 5 implies cos θ > 1. Physically, there is no partial wetting droplet and, hence, no equilibrium value of contact angle θ, although there will be a liquid film. Allowing eq 5 to be generalized to allow numerical values greater than unity for “cos θ” suggests a possible approach to using the Cassie equation for a component surface with a complete wetting surface chemistry. Here, we show that this leads to a physically meaningful description of the wetting of composite surfaces with both droplet and film-forming surface components.

In the remainder of this article, we use a minimization of surface free energy approach to derive a version of the Cassie equation formulated using spreading coefficients. We then show the consistency of our reformulated equation with Cassie’s original equation for the case where both surface components have negative spreading coefficients corresponding to droplet states. We also show that when one surface component has a positive spreading coefficient corresponding to a superspreading film state, there is a condition on the surface fraction ratio at which a transition from a droplet to a film state occurs on the composite surface. We then reinterpret literature data showing that such transitions exist and consider how recent experimental data can be reconciled with or provide challenges to the Cassie equation. Finally, we consider the extreme limit when droplets on composite surfaces exhibit complete non-wetting and argue that the strength of superhydrophobicity of such surfaces is not all equal.

## Theory—Cassie’s Law Reformulated

### Interfacial Energies for a Smooth Composite Surface

For simplicity, we consider here a composite surface with two surface components and use a two-dimensional model. However, the model can be generalized to multiple surface components and a three-dimensional model. We also follow the assumptions used in previous considerations of Cassie, Cassie–Baxter and Wenzel formulations, which assume the influence of patterns of wettability can be averaged out when considering minimum surface energy states for droplets. Such assumptions clearly break down when the droplet contact area is on similar length scales to any surface pattern or where strong pinning or faceting occurs.^10,13,27^

We now consider a smooth surface composed of two surface chemistries each with their own solid–liquid and solid–vapor interfacial energies. Since interfacial tensions are energies per unit area, we assume they are additive. We therefore write the corresponding interfacial energies of the composite surface in the vicinity of a three-phase contact line location *x* as
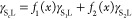
6and
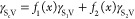
7where *f*_1_(*x*) and *f*_2_(*x*) are the local surface area fractions and satisfy *f*_1_(*x*) + *f*_2_(*x*) = 1. To determine whether a droplet on the composite surface is in local equilibrium, we consider a small change in position Δ*x* of the three-phase contact line at a location *x*. At the solid surface, this interchanges solid–vapor and solid–liquid interfacial energies, giving a change in interfacial energy of . In addition, the liquid–vapor interfacial area changes by Δ*x* cos θ_c_(*x*), where θ_c_(*x*) is the local value of the contact angle at the contact line, and so the associated change in liquid–vapor interfacial energy is γ_LV_Δ*x* cos θ_c_(*x*). The total change in surface free energy, Δ*E*, is then
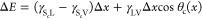
8which indicates that the contact line is at a local minimum in energy when Δ*E* = 0, i.e.,
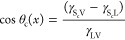
9

Equation 9 is in the form of Young’s law for a partially wetting droplet with an equilibrium contact, but, importantly, there has been no explicit requirement that a partially wetting droplet exists on either of the two component surfaces individually.

We now substitute eqs 6 and 7 into 9 and group the solid–liquid and solid–vapor interfacial tensions
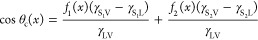
10

To obtain Cassie’s law (eq 1), we could require the combinations of interfacial tensions for each component surface to satisfy Young’s law, i.e.,
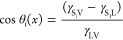
11

However, if we do that, we do not have to impose the additional condition that θ_*i*_(*x*) has to have a physical value corresponding to a partially wetting droplet. In principle, the combinations of interfacial tensions in eq 11 do not require the restriction −1 ≤ cos θ_*i*_(*x*) ≤ 1.

### Cassie’s Law Using Spreading Coefficients

To obtain a general case describing component surfaces with droplet- or film-forming wetting properties, we group interfacial tensions in eq 10 into combinations representing spreading coefficients, i.e.,
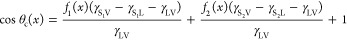
12

Using eq 4 for each component surface gives
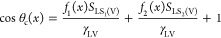
13or, more generally,
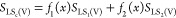
14In this reformulation of Cassie’s equation, the assumption that the wetting of a composite surface behaves as surface area-weighted averages for the solid–liquid and solid–vapor interfacial energies leads to a surface area-weighted average for the spreading coefficients. Equation 14 is a key result of this work and represents a generalized Cassie equation for composite surfaces; the equation can be simply extended to surface area-weighted averages of more than two component types.

**Figure 1:**
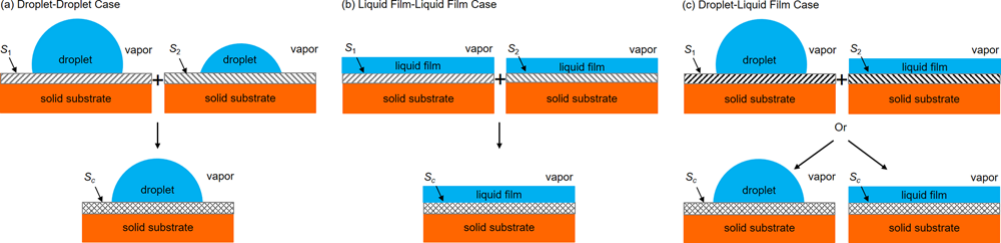
Three types of composite surfaces. (a) Two droplet-forming surface components, (b) two film-forming surface components, and (c) one droplet and one film-forming surface component.

There are three physical cases for the wetting properties of the component surfaces described by eq 14: (a) partial wetting for both (i.e., droplet-forming surfaces), (b) complete wetting for both surfaces, and (c) partial wetting for one surface and complete wetting for the other surface (i.e., a droplet-forming and a film-forming surface), (see Figure 1).

#### Surfaces Where Both Surface Components Are Partially Wetting

In this case, both spreading coefficients for the component surfaces are negative, i.e.,  and , and so, the spreading coefficient of the composite surface is also negative, i.e., . Physically, if a droplet on each component surface has a physically meaningful contact angle given by eq 11 (or equivalently eq 5), so does the droplet on the composite surface. Equation 14 could be written in the same form as eq 1 as a surface area fraction weighted average of cosines, with the surface area fractions being the appropriate values at the three-phase contact line of the droplet on the composite surface. However, there is also an interesting possibility of whether a composite surface might have a spreading coefficient corresponding to cos θ_c_(*x*) < −1 when the cosine is calculated using combinations of interfacial tensions, i.e.,
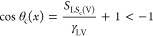
15

We consider this possibility, related to the question “*How superhydrophobic can a completely nonwetting surface be?”*, at the end of our Discussion section.

#### Surfaces Where Both Surface Components Are Completely Wetting

In this case, both spreading coefficients for the component surfaces are greater than or equal to zero, i.e.,  and , and so the spreading coefficient of the composite surface is also greater or equal to zero, i.e., . Physically, a droplet on each component surface would be a film and would not have a physically meaningful equilibrium contact angle given by eq 11 (or equivalently eq 5) unless the spreading coefficients were exactly equal to zero (in which case the contact angles would be 0°). Writing eq 14 in the same form as eq 1 as a surface area fraction weighted average of cosines with 0° for the two component surfaces would be a misleading characterization of the wetting properties of the composite surface. This is because different composite surfaces can have different values of the spreading coefficient, while each spreading coefficient remains positive. Increasingly positive spreading coefficients correspond to increasing differences in the interfacial energy difference per unit area between a bare surface and a liquid-coated surface and drive spreading (see eq 4). This has physical implications in, e.g., the driving of superspreading of a droplet from an out-of-equilibrium droplet state and the evolution of the dynamic contact angle.^28^ Allowing for superspreading, eq 1 would need to interpret the cosine of the contact angle as the physically meaningful quantity calculated through a combination of interfacial tensions and not through a measurable contact angle, i.e.,
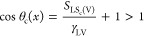
16

#### Surfaces Where One Component Is Partially Wetting and One Is Completely Wetting

In this case, we consider (without loss of generality) a composite surface where the first component surface is partial wetting, i.e., , and the second is complete wetting, i.e., . Here there is competition between droplet-forming and film-forming properties, and so, there will be specific surface fractions at which the composite surface will transform from supporting a droplet state with  to a film state with . Physically, this seems reasonable, as increasing the proportion of surface with film-forming properties compared to the proportion with droplet-forming properties should eventually overcome the droplet-forming tendencies of the composite surface. To consider the conditions under which a smooth composite surface transforms from droplet-forming to film-forming tendencies, in eq 14, we set *f*_2_ = *f*, *f*_1_ = 1 – *f* and use subscripts “*d*” (for droplet-forming) and “*f*” (for film-forming) for component surfaces 1 and 2, respectively, i.e.,
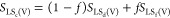
17

The threshold surface area fraction, *f*_T_, for a film to be induced on the composite surface is when the spreading coefficient for the composite surface increases to zero as the area fraction of the film-forming component of the surface is increased, i.e.,
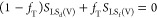
18

This gives a threshold value of the film-forming area fraction as
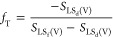
19

For composite surfaces with *f* < *f*_T_, we can replace two of the spreading coefficients in eq 17 by their contact angles, i.e.,
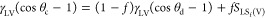
20

This can be re-written as
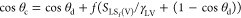
21and so, the threshold surface area fraction for film formation on the composite surface is given in terms of a measurable droplet contact angle, θ_d_, on the first surface component as
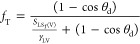
22

Using eq 5 to define a cos θ_f_ in terms of the interfacial tensions, this is equivalent to
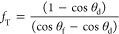
23

Experimentally, if it is possible to create a set of composite surfaces with different film-forming surface area fractions, *f*, and measure the contact angles on the composite surface, eq 21 allows the spreading coefficient for the film-forming surface component scaled by the liquid–vapor surface tension, *S*_Lf(V)_/γ_LV_, to be determined from the slope of a graph of cos θ_c_ versus *f*. Alternatively, this can be determined by identifying the surface area fraction for the transition from a droplet-forming to a film-forming composite surface. If we also know the liquid–vapor interfacial tension and the solid–liquid interfacial tension for the film-forming component of the surface, the solid–vapor interfacial tension for the film-forming component of the surface could be calculated.

## Discussion of Literature Data

### Mixed Self-Assembled Monolayer Composite Surface

Historically, the experiments by Laibinis and Whitesides^23^ on the wetting by droplets of water of self-assembled mixed monolayers of the hydrophobic thiol HS(CH_2_)_11_CH_3_ and the hydrophilic thiol HS(CH_2_)_11_OH deposited from solutions using ethanol or isooctane onto freshly deposited evaporated copper, silver, and gold films are regarded as establishing the validity of the original Cassie equation.^24^ From our perspective, these are particularly interesting datasets because they use a hydrophobic methyl termination and a hydrophilic hydroxyl termination for the two surface components. Moreover, the surface compositions, χ^Surf^, were determined by X-ray photoelectron spectroscopy (XPS) and shown to vary from 0 to 1 for the hydroxyl surface component, χ_OH_^Surf^. These surfaces were prepared from a mixed solution of the two thiols, i.e., they did not have a specific surface pattern with distinct hydrophobic and hydrophilic regions. Thus, the assumption in the Cassie equation that the length scale of any surface patterning is less than the scale of a droplet contact area is likely to be satisfied unless there is significant natural clustering into islands occurring in the self-assembly process. In applying the Cassie model, it is assumed the interfacial energies are additive, and while this is accepted for macroscopic models of capillarity, it may be challenged at a molecular level. The water contact angle measurements in the data were also reported as advancing, θ_a_, and receding, θ_r_, contact angle measurements rather than simply static contact angles.

**Figure 2:**
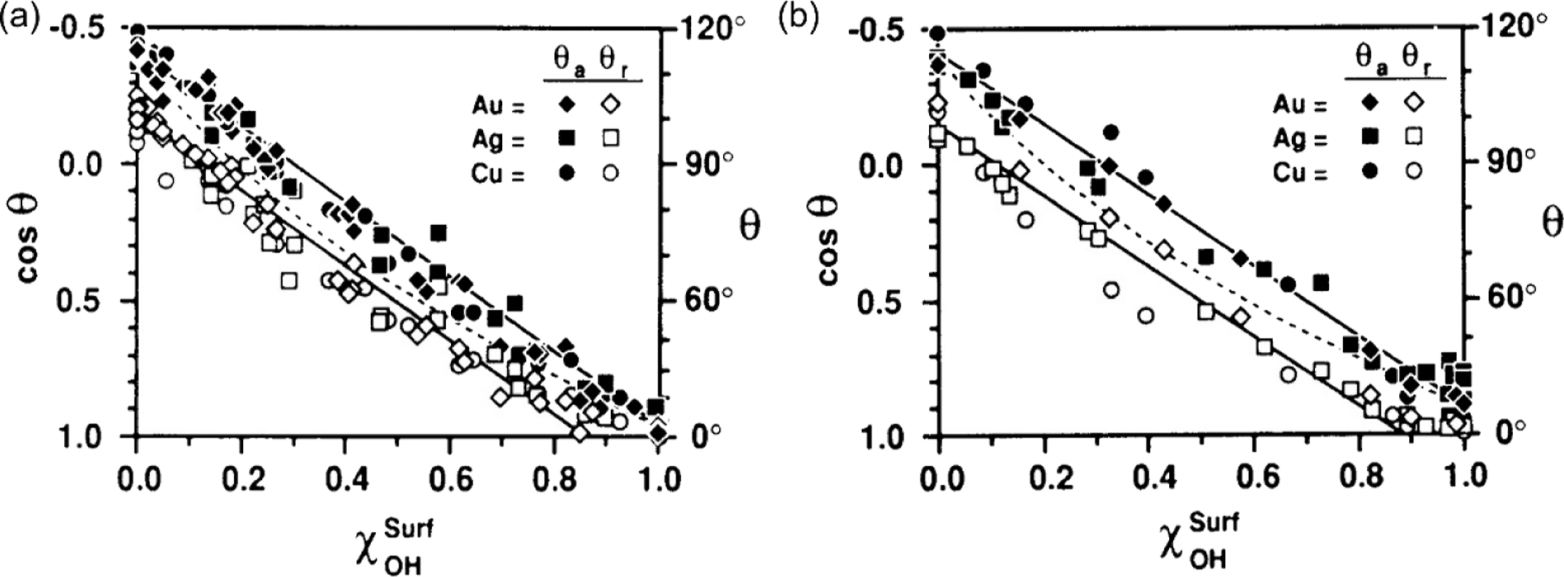
Data for contact angles of water droplets on mixed self-assembled monolayers (adapted from Laibinis and Whitesides.^23^ Copyright 1992 by the American Chemical Society). Comparison of monolayers adsorbed on copper, silver, and gold from mixtures of HS(CH_2_)_11_OH and HS(CH_2_)_11_CH_3_ dissolved in (a) ethanol after 2 h exposure, and (b) isooctane after 1 h exposure. Surface compositions were determined by XPS and assumed to be equivalent to surface area fractions.

**Figure 3:**
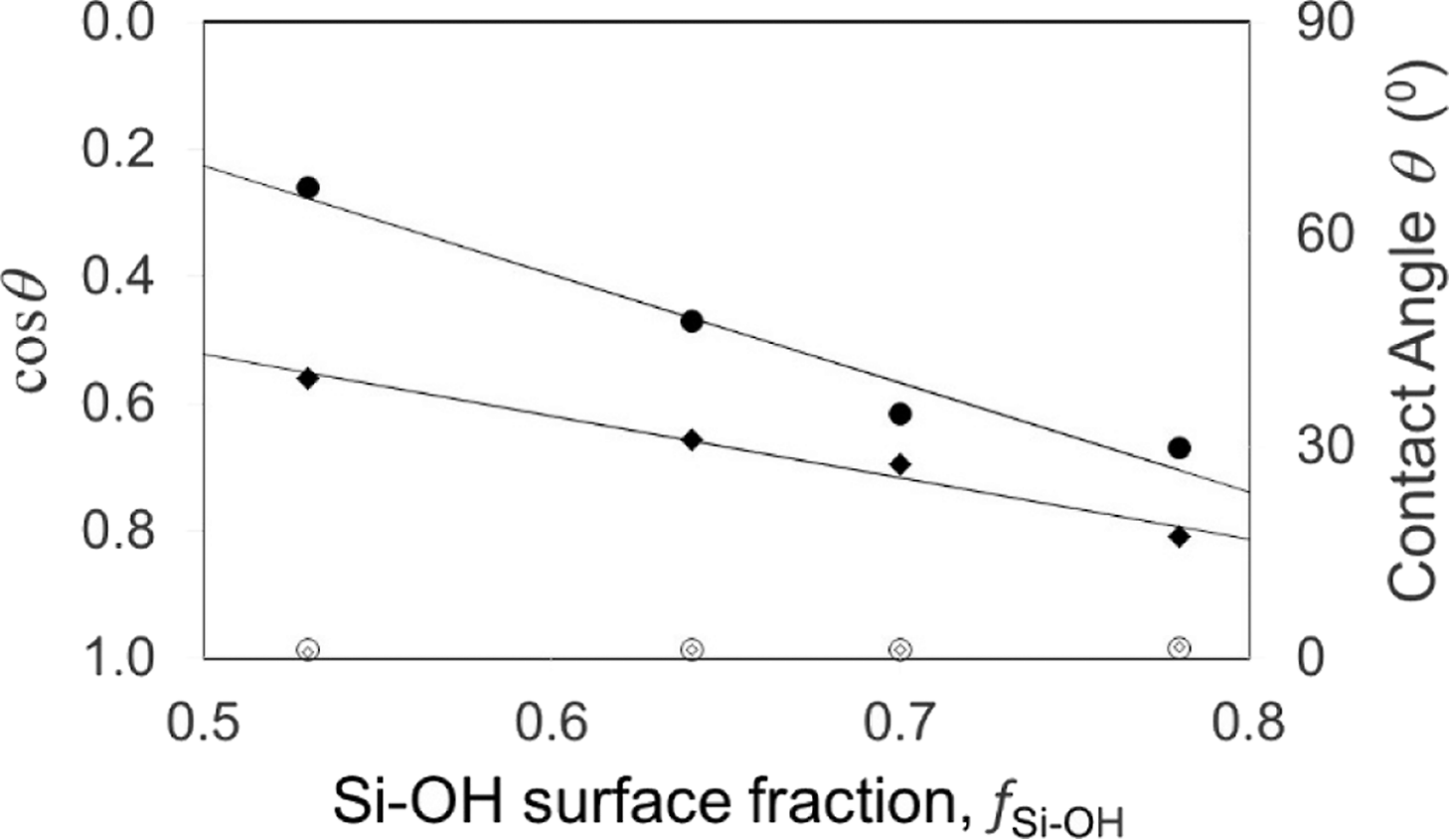
Advancing and receding contact angle data for water droplets on checkerboard-like composite surfaces (data from Becher-Nienhaus et al.^25^). Advancing contact angles on *D*_4_^H^/Si–OH surfaces are shown as solid diamond symbols (◆◆◆) and on ODS/Si–OH surfaces are shown as solid circle symbols (●●●). The corresponding data for receding contact angles is shown using open symbols. The solid lines are fits of eq 25 to the data for advancing contact angles.

Figure 2 reproduces the two key graphs (Figures 2c and 3c) from the original paper by Laibinis and Whitesides.^23^ These data show the measured advancing contact angles (solid symbols) and receding contact angles (open symbols) for water droplets on mixed monolayers deposited from mixtures of −CH_3_- and −CH_2_OH-terminated thiols in ethanol or iso-octane, respectively, onto gold, silver, and copper films and their dependence on the hydroxyl surface composition, χ_OH_^Surf^. The authors of ref (23) comment that they assume that the fractional surface areas of hydroxyl and methyl groups are equivalent to the surface compositions obtained by XPS in the systems they report. The solid lines on each figure are the original authors’ illustration that Cassie’s equation (eq 1) was a better description of the data than an alternative equation suggested by Israelachvili and Gee^29^ (dashed lines).

To be explicit about the relationship to Cassie’s equation (eq 1), θ_2_ = θ_OH_ is the contact angle on a surface with 100% hydroxyl groups, , is the contact angle on a surface with 100% methyl, and the surface area fractions of the component surfaces are *f*_2_ = χ_OH_^Surf^ and , i.e.,
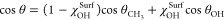
24In eq 24, the contact angles are either the advancing or the receding contact angles. Laibinis and Whitesides did not state that the solid lines through the data points were best fits through some specific range of the data points or give specific fitting equations for these lines. We therefore assume they were visual guides to the eye, showing that linear relationships and the expected trends with composition justified the Cassie equation (eq 1 or, equivalently, eq 24) as the most consistent description of the data. We therefore re-examined the solid lines in Figure 2 and determined the values of  and cos θ_OH_ for eq 24 for each solid line (Table 1); these give contact angles entirely consistent with the values of water contact angles for monolayers of HS(CH_2_)_11_CH_3_ and HS(CH_2_)_11_OH reported in Table 1 of Laibinis and Whitesides.^23^ The first column in Table 1 identifies the solvent in the solution from which the self-assembled monolayer (SAM) was deposited to create the surface. The receding contact angle data can only be fitted using cos θ_OH_^r^ > 1, corresponding to a non-physical receding contact angle on the hydroxyl surface component. Visually, in Figure 2, the solid lines through the receding contact angle data for the composite surfaces cross the *y*-axis at cos θ = 1 when the surface composition of the hydrophilic hydroxyl surface component is χ_OH_^Surf^ = 0.86 ± 0.02. Thus, increasing the hydrophilic hydroxyl-group terminated surface component eventually has a stronger influence than the hydrophobic methyl-group terminated surface component and transforms the mixed SAM surface into one with film-forming properties. Moreover, it is able to do so without needing to have a 100% hydrophilic hydroxyl-group terminated surface because the spreading coefficient *S*_LOH(V)_ > 0 for receding experiments.

**Table 1:** Parameters Required in the Cassie Equation to Fit Laibinis and Whitesides^23^ Data for Wetting of Mixed Self-Assembled Monolayers by Droplets of Water

SAM solution	contact angle type	cos θ_CH_3__	cos θ_OH_	cos θ_CH_3__ (deg)	θ_OH_ (deg)	threshold χ_OH_^Surf^
ethanol	advancing	–0.42 ± 0.02	0.96 ± 0.01	115 ± 1	16 ± 2	1.03 ± 0.02
ethanol	receding	–0.18 ± 0.02	1.19 ± 0.01	100 ± 1	N/A	0.86 ± 0.02
isooctane	advancing	–0.41 ± 0.01	0.88 ± 0.01	114 ± 1	28 ± 2	1.09 ± 0.02
isooctane	receding	–0.14 ± 0.01	1.13 ± 0.01	98 ± 1	N/A	0.90 ± 0.02

### Lithographically Patterned Composite Surfaces with a Superhydrophilic Component

We now consider the recent data reported by Becher-Nienhaus et al.^25^ which used smooth checkerboard-like micropatterned hydrophobic/(super)hydrophilic surfaces and which they suggested was not well-described by the Cassie equation. The pattern sizes were 2, 5, 10, and 20 μm, with regions of matching/mismatching CAH created using chemisorption and photopatterning of monolayers. Their experiments involved binary composite surfaces using combinations of four surface chemistries: (i) monolayers of hydrophobic 1,3,5,7-tetramethylcyclotetrasiloxane on silicon (*D*_4_^H^, low CAH), (ii) monolayers of octadecyltrimethoxysilane on silicon (ODS, high CAH), (iii) superhydrophilic Si–OH by photodecomposition of *D*_4_^H^ or ODS monolayers on silicon, and (iv) hydrophilic 2-[methoxy(polyethyleneoxy)6-9propyl]-trimethoxysilane monolayers on silicon (PEG, low CAH).

We first consider the experimental data for the binary composite surfaces involving the superhydrophilic Si–OH surface component with the two hydrophobic surface components (*D*_4_^H^ or ODS). The data published by Becher-Nienhaus et al.^25^ is reproduced in Table 2 with two additional columns we have added to show the cosine values of the experimentally obtained advancing and receding contact angles. To calculate values using Cassie’s (eq 1), Becher-Nienhaus et al. used advancing contact angles of θ_a_ = 103° ± 2°, θ_a_ = 110° ± 3° and θ_a_ = 0°, and receding contact angles of θ_r_ = 98° ± 3°, θ_r_ = 99° ± 3° and θ_r_ = 0° for *D*_4_^H^, ODS, and Si–OH, respectively. The use of advancing and receding contact angles of 0° for the Si–OH surfaces was based on the observation that spreading occurs. However, this would mean that the spreading coefficient for the surface was precisely equal to unity, i.e., *S*_LSi–OH(V)_ = 0, which is unlikely to be the case. We therefore believe the differences between their calculated contact angles using Cassie’s equation (eq 1) and the measured ones for composite surfaces using Si–OH arise from an (incorrect) implicit assumption that the spreading coefficient on Si–OH is zero rather than greater or equal to zero. An important consequence of this is that it reduces the data set against which comparisons to the Cassie law, eq 1, can be made and hence confidence in conclusions that might be deduced.

**Table 2:** Data from Binary Micropatterned Surfaces by Becher-Nienhaus et al^25^

	θ_a_/θ_r_ (deg)					
	*D*_4_^H^/Si–OH			ODS/Si–OH		
size (μm)	calcd ref (25)	exptl	cos θ_a_/cos θ_r_	calcd ref (25)	exptl	cos θ_a_/cos θ_r_
2	43/41	(36 ± 2)/(11 ± 2)	(0.81 ± 0.02)/(0.982 ± 0.007)	45/42	(48 ± 4)/(10 ± 3)	(0.67 ± 0.05)/(0.985 ± 0.006)
5	51/49	(46 ± 3)/(9 ± 2)	(0.70 ± 0.03)/(0.988 ± 0.006)	53/49	(52 ± 3)/(9 ± 2)	(0.62 ± 0.05)/(0.988 ± 0.006)
10	56/54	(49 ± 2)/(9 ± 2)	(0.66 ± 0.03)/(0.988 ± 0.006)	59/54	(62 ± 2)/(9 ± 2)	(0.47 ± 0.03)/ (0.988 ± 0.006)
20	65/62	(56 ± 2)/(8 ± 2)	(0.56 ± 0.03)/(0.990 ± 0.005)	68/63	(75 ± 3)/(9 ± 2)	(0.26 ± 0.05)/ (0.988 ± 0.006)

We now focus on whether the trends in the advancing and receding contact angles on each composite surface using Si–OH follow expectations from Cassie’s equation. To do so, we consider whether data can be fitted to a linear equation analogous to eq 24, i.e.,
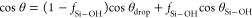
25where θ_drop_ is the contact angle on the other surface component, i.e.,  or θ_ODS_^H^. Figure 3 shows the fits of eq 25 to the experimental data for the advancing contact angles on *D*_4_^H^/Si–OH and ODS/Si–OH (lines through the solid symbols). This shows a reasonable linearity consistent with expectations from eq 25, albeit with the limited data set of only four Si–OH surface fractions. Moreover, these fits reproduce the measured advancing contact angles on *D*_4_^H^/Si–OH surfaces to within 2° and on ODS/Si–OH surfaces to within 3°, i.e., agreement is within the reported experimental error on the measured values. From the fits, we also deduce that the advancing contact angles of the partially wetting surface components in the composite *D*_4_^H^/Si–OH and ODS/Si–OH surfaces are  and θ_ODS_^H^ = 129°, respectively. These are different from the values of  and θ_ODS_^H^ = 110 ± 3°, respectively, reported by Becher-Nienhaus et al.^25^ It can be argued that the fits here give an unrealistically high advancing contact angle for θ_ODS_^H^ unless the surface has some roughness. However, given the limitation of having only four data points, our main comment is that the trends are consistent with eq 25. Another possible explanation of this difference from the experimental perspective is that the values reported in the paper were measured on complete monolayers, which had not been subsequently subjected to the masking and exposure process used to photo-decompose areas to create the Si–OH component of the composite surfaces. We cannot therefore be certain that these reported values for the advancing (or receding) contact angles on the *D*_4_^H^ and ODS surfaces reflect their values on the *D*_4_^H^ and ODS surface components of the composite surfaces. The challenge this discussion highlights is that a key challenge is to achieve a richer data set to allow more reliable comparisons. Figure 3 also shows the fits to the advancing contact angle data give cos θ_Si–OH_ = 1.01 and cos θ_Si–OH_ = 1.08 for the Si–OH of the *D*_4_^H^/Si–OH and ODS/Si–OH surfaces, respectively, thus confirming the Si–OH surface components have superspreading properties, i.e., spreading coefficients above the threshold of 1 necessary for complete wetting.

We now consider the receding contact angle data (open diamonds and open circles) in Figure 3. For these data points, there is no obvious dependence on the Si–OH surface fraction. The measured receding contact angles vary between 9 and 11° which corresponds to cos θ_r_ varying from 0.98 to 0.99, i.e., essentially cos θ_r_ is constant and ∼1 (see Table 2). It is therefore plausible that these composite surfaces were behaving consistently with a surface whose surface fraction of the complete wetting component (Si–OH) was beyond the threshold value for complete wetting despite the small, finite receding contact angles reported.

### Lithographically Patterned Composite Surfaces with a Partially Wetting Hydrophilic Component

**Figure 4:**
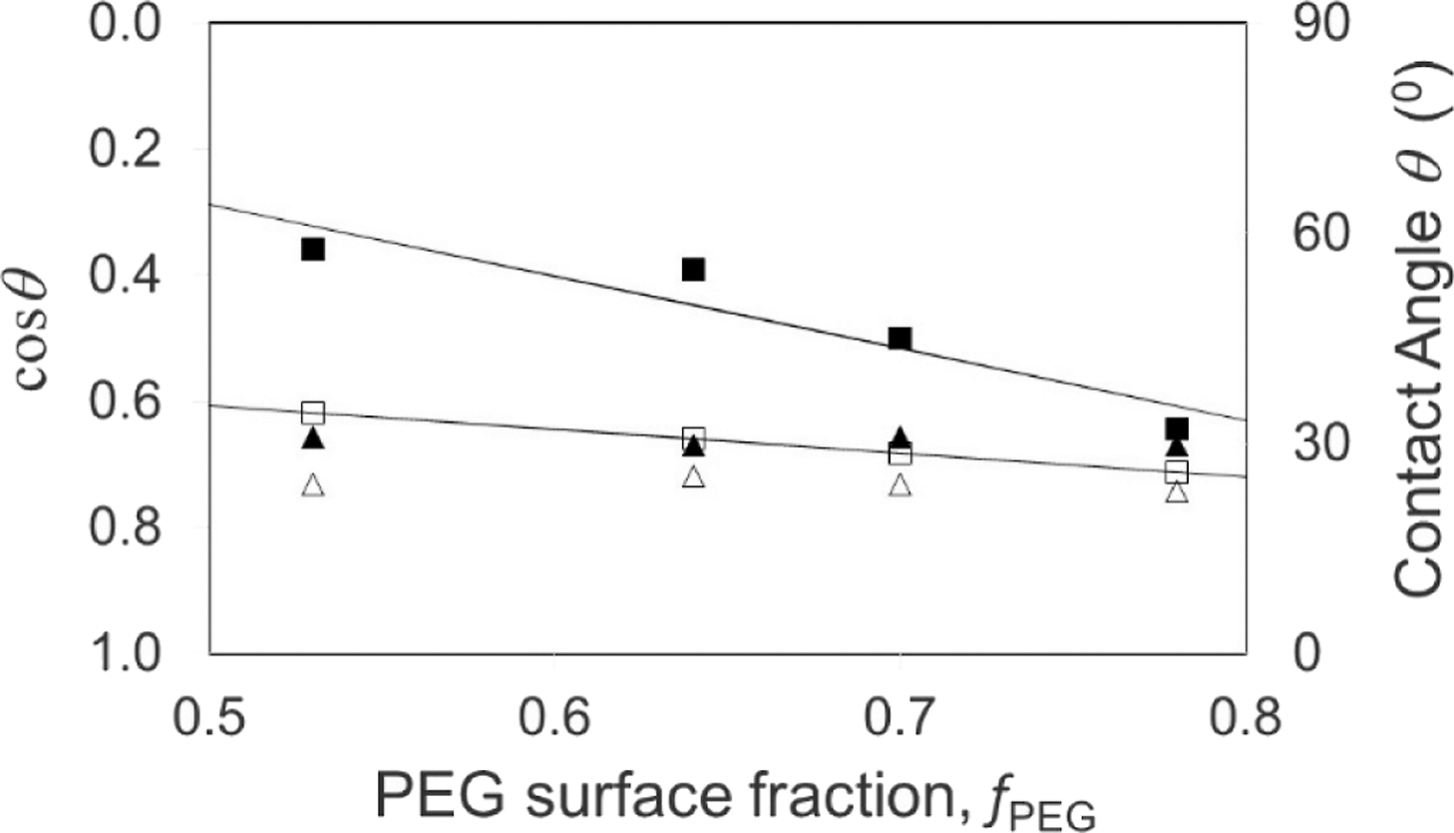
Advancing and receding contact angle data for water droplets on checkerboard-like composite surfaces (data from Becher-Nienhaus et al.^25^). Advancing contact angles on *D*_4_^H^/PEG surfaces are shown as solid triangle symbols (▲▲▲) and on ODS/PEG surfaces are shown as solid square symbols (■■■). The corresponding data for receding contact angles is shown using open symbols. The solid lines are fits of eq 26 to the data for advancing and receding contact angles on the ODS/PEG surfaces.

A similar consideration of data can be performed for the advancing and receding contact angles on each composite surface using PEG. In this case, the relevant equation to consider is
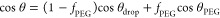
26where θ_drop_ is the contact angle on the other surface component, i.e.,  or θ_ODS_^H^. In Figure 4, solid square symbols show the advancing contact angles and open symbols show the receding contact angles on the ODS/PEG surface. The solid lines show fits through these data points using θ_ODS_ = 106° and θ_PEG_ = 31° for the advancing contact angle data and are θ_ODS_ = 65° and θ_PEG_ = 37° for the advancing contact angle data on the ODS/PEG surface. This compares to advancing contact angles of θ_ODS_ = 110 ± 3° and θ_PEG_ = 40 ± 1° and receding contact angles of θ_ODS_ = 99 ± 3° and θ_PEG_ = 36 ± 1° on monolayer surfaces. We conclude from this data that Cassie’s equation may describe the data, but that the contact angle for the hydrophobic ODS appears much reduced compared to its value from a uniform monolayer. In contrast, the *D*_4_^H^/PEG composite surface is not well-described by Cassie’s equation. In Figure 4, filled triangle symbols show the advancing contact angles, and open triangle symbols show the receding contact angles on the *D*_4_^H^/PEG composite surface. For this surface, the data appears insensitive to the PEG surface area fraction, which is inconsistent with Cassie’s equation. From the experimental perspective, one possible explanation is that the method of preparation of composite samples involving PEG may have led to changes in the ODS/*D*_4_^H^ surface components. In particular, masking ODS/*D*_4_^H^ monolayers, photo-decomposing selected regions to create SiOH regions, and then chemisorbing PEG may have changed the ODS/*D*_4_^H^ regions. For example, PEG molecules could physisorb on top of the *D*_4_^H^ and ODS monolayers or chemisorb on empty patches of the incomplete monolayers of *D*_4_^H^ and ODS (or on parts damaged during the patterning through the photomask), and therefore the contact angle on the hydrophobic parts of the patterns could be lower than expected based on the non-patterned *D*_4_^H^.

### Composite Surfaces That Are Beyond the Threshold for Superhydrophobicity

Our reformulation of Cassie’s equation using spreading coefficients (eq 14) illustrates that it is possible, in principle, to consider composite surfaces with a wide range of values of cos θ_c_, which do not need to all correspond to physical values of contact angles in the range 0° ≤ θ_c_ < 180°. In particular, when one or both surfaces have positive spreading coefficients, a composite surface can be created with a positive spreading coefficient, i.e., film-forming properties with cos θ_c_ > 1. Such a situation is possible because suitable combinations of surface chemistry and liquids exist. In contrast, the other extreme with cos θ_c_ < −1 does not appear physically possible for water on a smooth, heterogeneous surface in air (or vapor) because hydrophobic −CF_3_-terminated surface coatings tend to have static contact angles around or below 120°. To achieve highly non-wetting surfaces with contact angles approaching 180°, i.e., cos θ_c_ → −1, either nano/micro-scale topographic structure is used to amplify the effect of hydrophobic surface chemistry into superhydrophobicity^6−8^ or a Leidenfrost effect vapor layer is used.^30^

Cassie and Baxter’s work in 1944, which preceded the development of eq 1 for heterogeneous smooth composite surfaces, considered a (superhydrophobic) model for porous surfaces using water on a parallel array of fibers.^3^ In this case, the wetting on the solid portion of the surface includes a Wenzel roughness factor *r* which transforms the contact angle from its value on a smooth surface into a new value
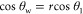
27

Thus, if surface component 1 is a rough surface for which the liquid remains in contact at all points (i.e., in a Wenzel state), and surface 2 is a smooth surface, Cassie’s equation becomes^31,32^
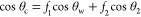
28where *f*_1_ + *f*_2_ = 1. To consider a superhydrophobic surface, we set surface component 2 as air, *f*_2_ = *f*_LV_ as the liquid–vapor surface fraction and θ_2_ = 180°, so that eq 28 becomes
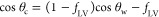
29

When the value of cos θ_w_ corresponds to a physical contact angle, eq 29 predicts an approach to complete non-wetting, i.e., θ_c_ → 180°, only occurs as the liquid–vapor surface fraction tends to unity, i.e., *f*_LV_ → 1. This corresponds to a suspended droplet in a superhydrophobic state. However, in principle, cos θ_c_ < −1 is possible when the surface roughness of the solid component satisfies , where θ_s_ is the contact angle on the smooth solid surface. It therefore appears that rough hydrophobic solids in the Wenzel state, combined with a larger-scale texture that suspends a droplet across air gaps, could provide surfaces that display complete non-wetting (apparent contact angles of 180°) but should not be regarded as equivalent to each other. For these surfaces, increasing roughness for the solid component allows the surfaces to be classified based on their overall values of effective spreading coefficients. We also note that while it is a common experimental observation that droplets in Wenzel states suffer from contact line pinning, it is now possible to create slippery Wenzel states so that a droplet on these rough-textured surfaces could remain entirely mobile.^33^

## Conclusions

In this work, we have developed a description of the wetting properties of smooth composite surfaces with droplet- and film-forming surface components. We have reformulated Cassie’s equation using spreading coefficients so that the overall spreading coefficient on a composite surface is a surface area-weighted average of the spreading coefficients on the component surfaces, where the area averages are evaluated at the contact line (see eq 14). We have shown that Cassie’s original equation can be generalized by defining cos θ using combinations of interfacial tensions and allowing it to include values that do not correspond to measurable contact angles but which are meaningful in classifying film-forming surfaces with different spreading coefficients. We have also shown there is a threshold surface area fraction for the superspreading (film-forming) component for a composite surface with a partial wetting (droplet-forming) surface component at which a film will be created (see eqs 19 and 22). We have further shown how this enables the spreading coefficient for superspreading surface chemistry to be obtained from contact angle measurements by using composite surfaces with increasing fractions of the superspreading component. These ideas have been tested against literature data and have been used to explain aspects of that data not previously commented upon. Finally, we have discussed the case of a composite surface with spreading coefficients, which can be interpreted as a surface beyond complete non-wetting (more than superhydrophobic). Our work provides a conceptual framework for the wetting properties of composite surfaces composed of both droplet and film-forming surface components.
